# Supplementary material for: An effect of serotonergic stimulation on learning rates for rewards apparent after long intertrial intervals
Source: Nat Commun. 2018 Jun 26;9:2477. doi: 10.1038/s41467-018-04840-2 (PMC6018802; doi:10.1038/s41467-018-04840-2)
Supplement: Supplementary file 1 — Supplementary Information [file 41467_2018_4840_MOESM1_ESM.pdf]

An effect of serontonergic stimulation on learning rates for rewards apparent after long intertrial intervals

Kiyohito Iigaya, et al.

Supplementary figures

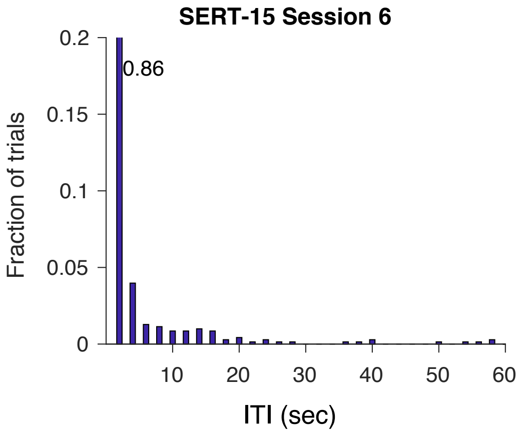

Supplementary Figure 1: The distribution of ITIs for SERT-Cre mice 15 in session 6, the same session as in Figure 1 d,f. The number located next to the tallest bar indicates the mean value of the bar, as the y-axis is cut at 0.2.

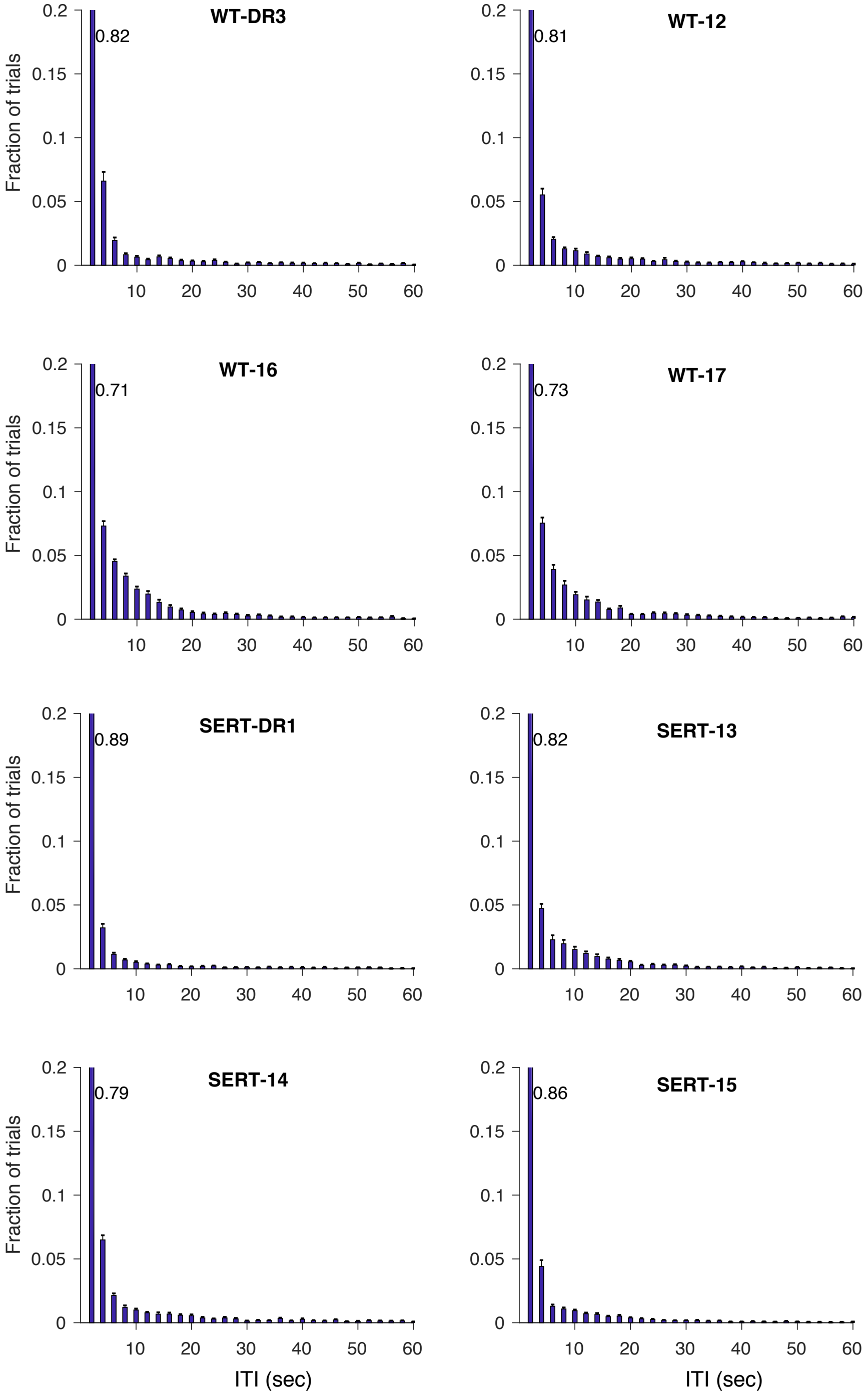

Supplementary Figure 2: The distribution of ITIs for all subjects. The error bars indicate the mean $\pm$ SEM of sessions. The numbers located next to the tallest bars indicate the mean values of the bars, as the y-axis is cut at 0.2.

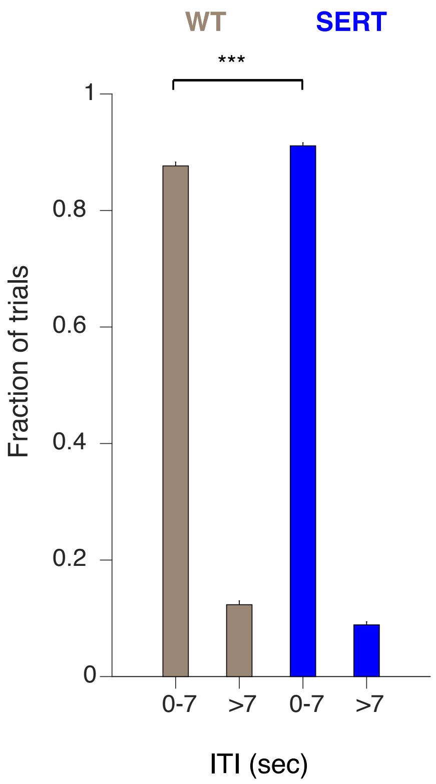

Supplementary Figure 3: The distribution of ITIs. The proportions of short (≤ 7 sec) ITI trials and long (> 7 sec) ITI trials were significantly different for both WT (left) and SERT-Cre (right) mice. The difference between WT and SERT-Cre mice was also significant, though the optogenetic stimulation itself did not change the subsequent ITIs (see **Figure 4**). The error bars indicate the mean $\pm$ SEM of sessions ($n = 32$).

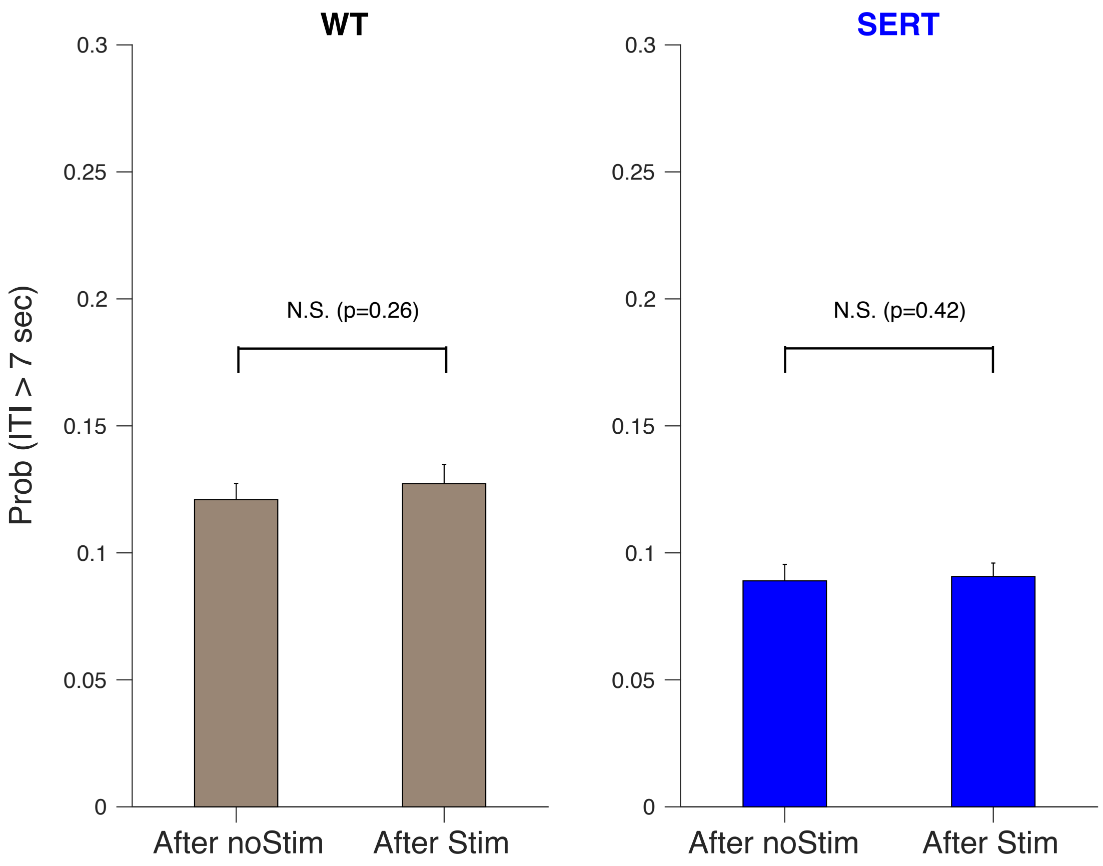

Supplementary Figure 4: Probability that the ITI is longer than 7 sec, following a photo-, or no photo-, stimulation. Stimulation does not significantly increase the chance of creating a long ITI event.

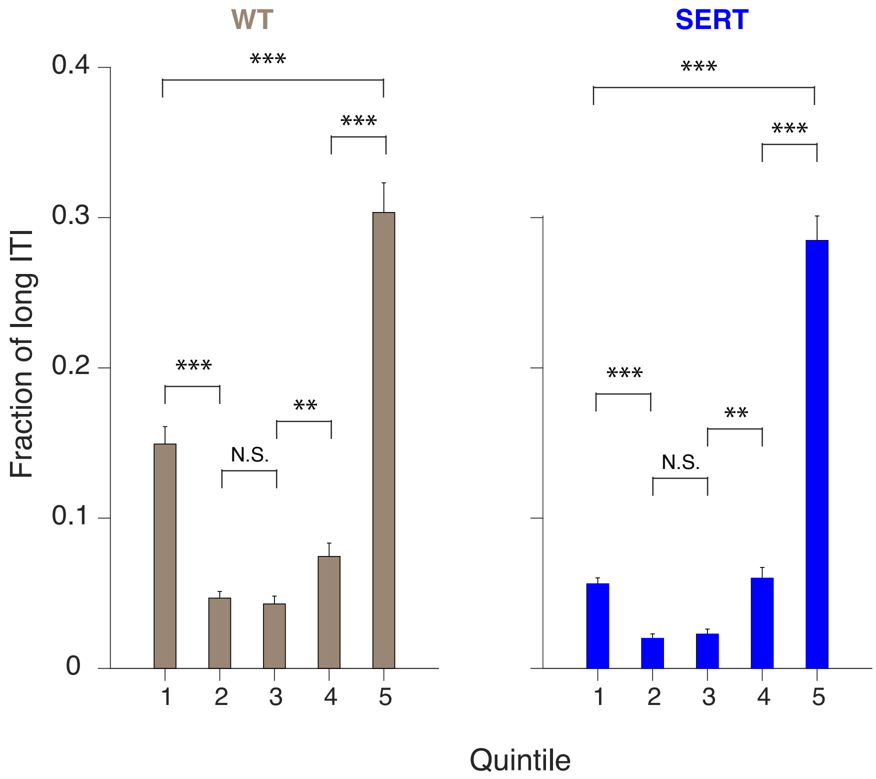

Supplementary Figure 5: The fractions of long ITI trials in quintiles (containing equal numbers) of trials within sessions for wild-type (left; grey) and SERT (right; blue) mice. The error bars indicate the mean $\pm$ SEM. The difference between the first and the second quintile ($p < 0.001$, permutation test), between the third and the fourth quintile ($p < 0.01$, permutation test), between the fourth and the fifth quintile ($p < 0.001$, permutation test), between the first and the fifth quintile ($p < 0.001$, permutation test) are significant within WT and SERT-Cre mice, respectively. The error bars indicate the mean $\pm$ SEM of sessions ($n = 32$)

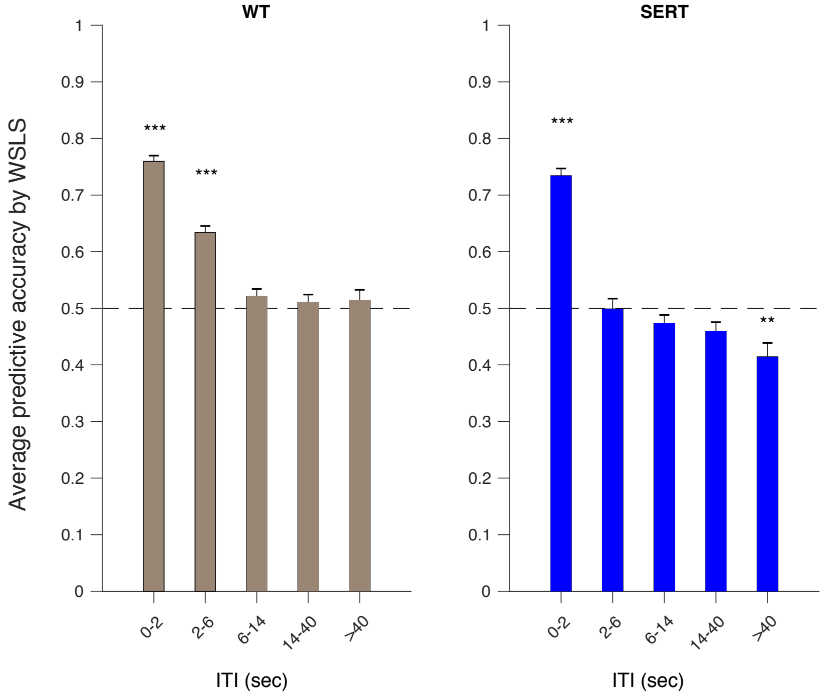

Supplementary Figure 6: Average predictive accuracies of win-stay lose-switch (WSLS) model for choices following different ITIs. Choices following shorter ITIs (smaller values in x axis) were well-predicted, but choices following longer ITIs (greater values in x axis) were not predicted by this simple model. The stars indicates the significance of the accuracy being different from chance, tested by t-tests. The error bars indicate the mean $\pm$ SEM of sessions $(n = 32)$

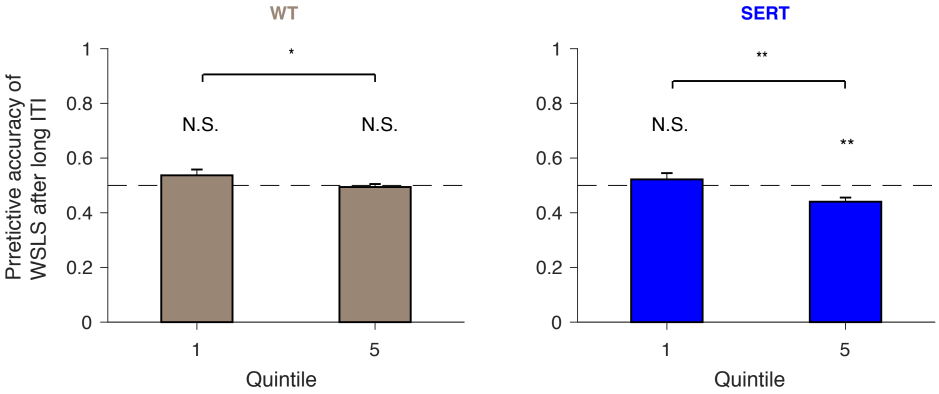

Supplementary Figure 7: Predictive accuracy of win-stay lose-switch (WSLS) model for choices following long ITIs in the 1st, and the last, quintiles of sessions. The predictive accuracy was not significantly larger than chance for both mice groups, though it was slightly, but significantly, lower than chance for the 5th quintile in SERT-Cre mice ($p < 0.01$, t-test). The difference between the first quintile and the 5th quintile was mildly significant ($p < 0.05$, permutation test) for WT mice, and it was significant ($p < 0.01$, permutation test) for SERT-Cre mice. The error bars indicate the mean $\pm$ SEM of sessions ($n = 32$)

Fitted to **long ITI trials**

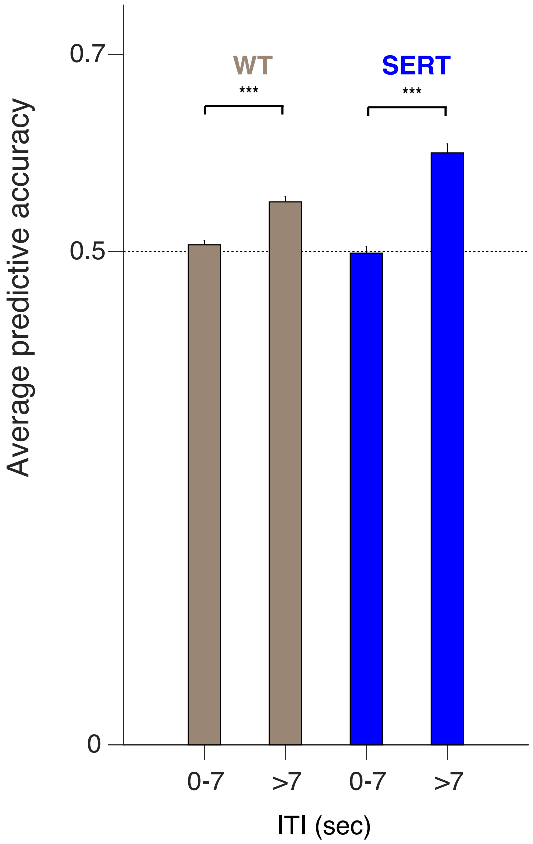

Supplementary Figure 8: Average predictive accuracies of the existing model (the same as in **Figure 1(g)**); but now the parameter fits were constrained only by choices following long ITIs. Choices following short ITIs ($\leq$ 7 sec) were not well-predicted by the model, while choices following long ITIs ($>$ 7 sec) were better predicted. The difference between short and long ITIs was significant for both WT and SERT mice [permutation test. $p < 0.001$ indicated by three stars.]. The error bars indicate the mean $\pm$ SEM of sessions ($n = 32$)

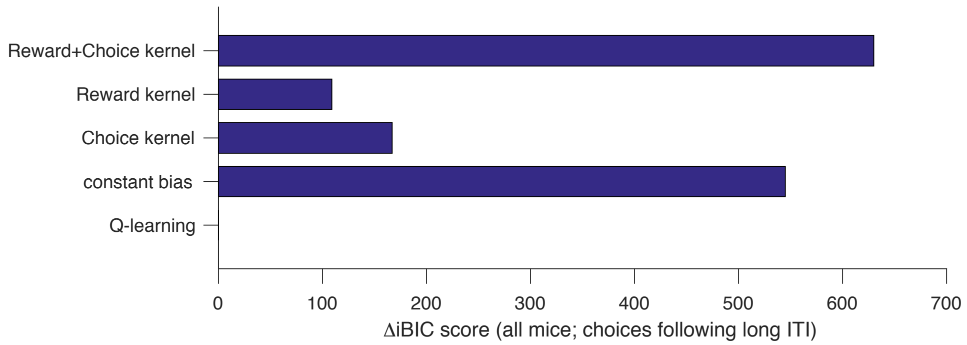

Supplementary Figure 9: Model comparison for choices following long ITIs, based on integrated Bayesian Information Criterion (iBIC). Q-learning model outperforms the other models. The previously validated model, Reward + Choice kernel model (top), performs poorly for choices following long ITIs.

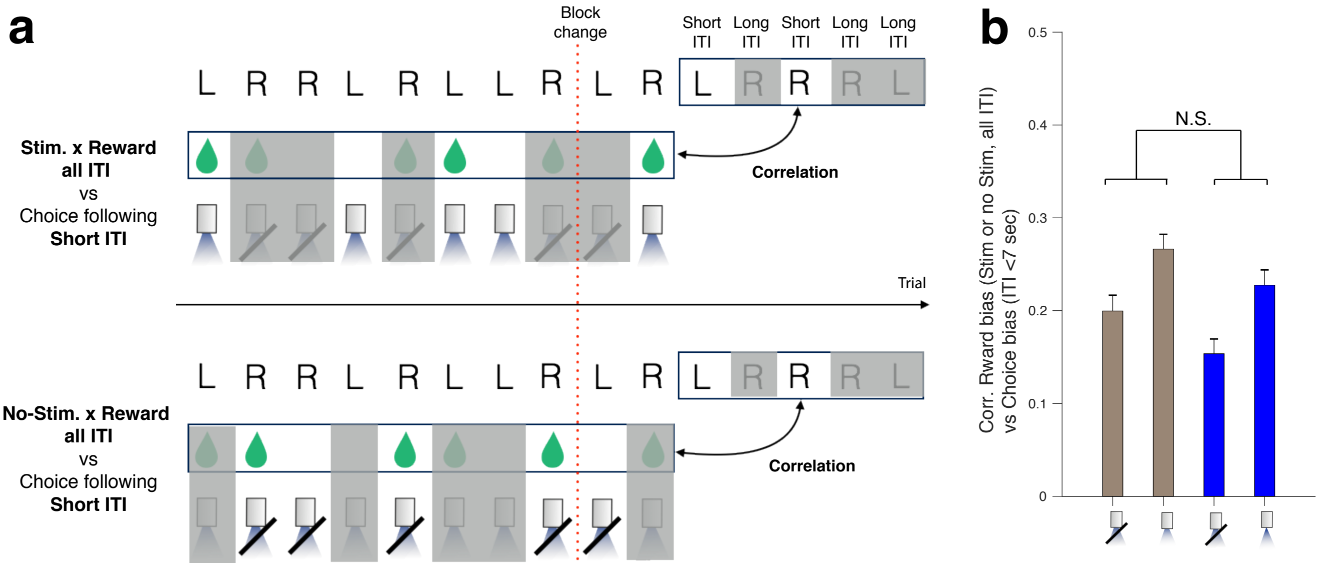

Supplementary Figure 10: The impact of reward history on choices following short ITIs did not show effects of optogenetic stimulation. (**a**) Schematics of analysis. It is the same as Figure 2a, except that choices follwoing short ITIs were now analyzed. (**b**) Results. The x-axis indicates if the reward bias was computed over trials with or without photo-stimulations. Due to the experimental bias of stimulation and reward probability, the correlation appears to be larger when stimulation is on for both groups; however, the difference between WT and SERT-Cre was not significant. The error bars indicate the mean ± SEM of sessions.

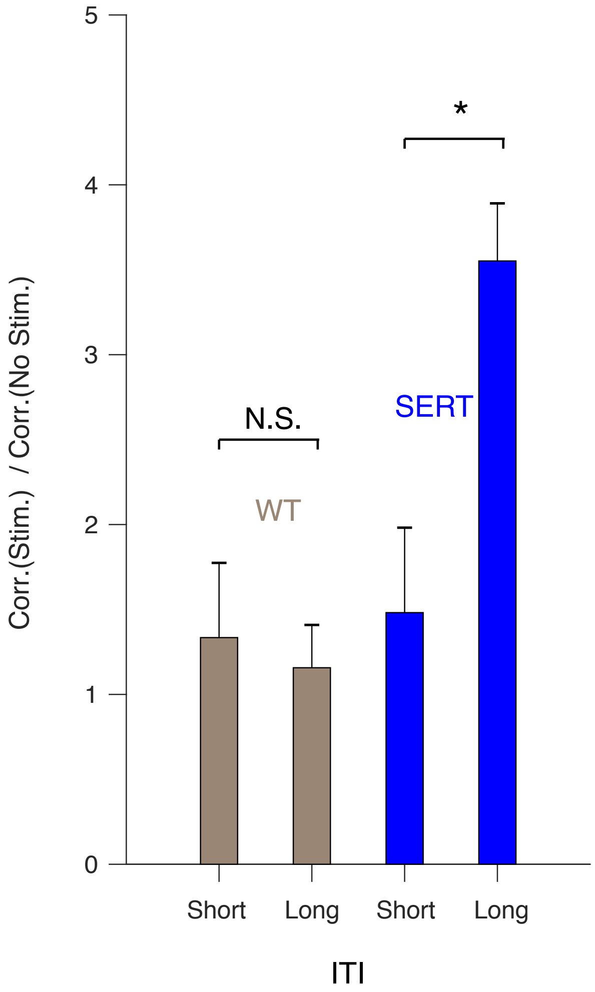

Supplementary Figure 11: The impact of reward history on choice was more strongly seen in choices following long ITIs than ones following short ITIs in SERT-Cre mice, while it was not the case in WT mice. The x-axis indicates if the correlation was computed for choices following short or long ITIs. The y axis indicates the ratio of the correlation between reward bias and choice bias computed over trials with photo-stimulations to the correlation computed over trials without photo-stimulations. The difference between the short and the long ITI conditions in SERT-Cre mice was significant (permutation test; $p < 0.05$), while the difference in WT mice was not significant. The error bars indicate the mean $\pm$ SEM of sessions ($n = 32$)

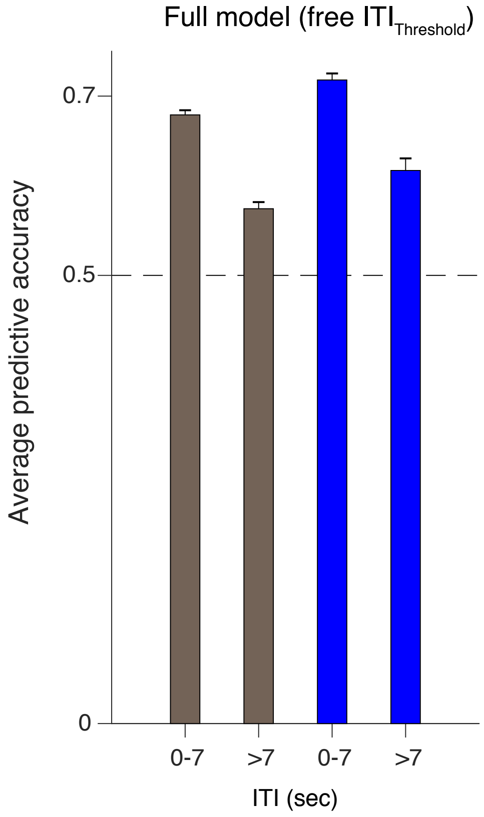

Supplementary Figure 12: Average predictive accuracies of the full model for short and long ITIs. Choices were well-predicted by the model. The error bars indicate the mean ± SEM of sessions ($n = 32$)

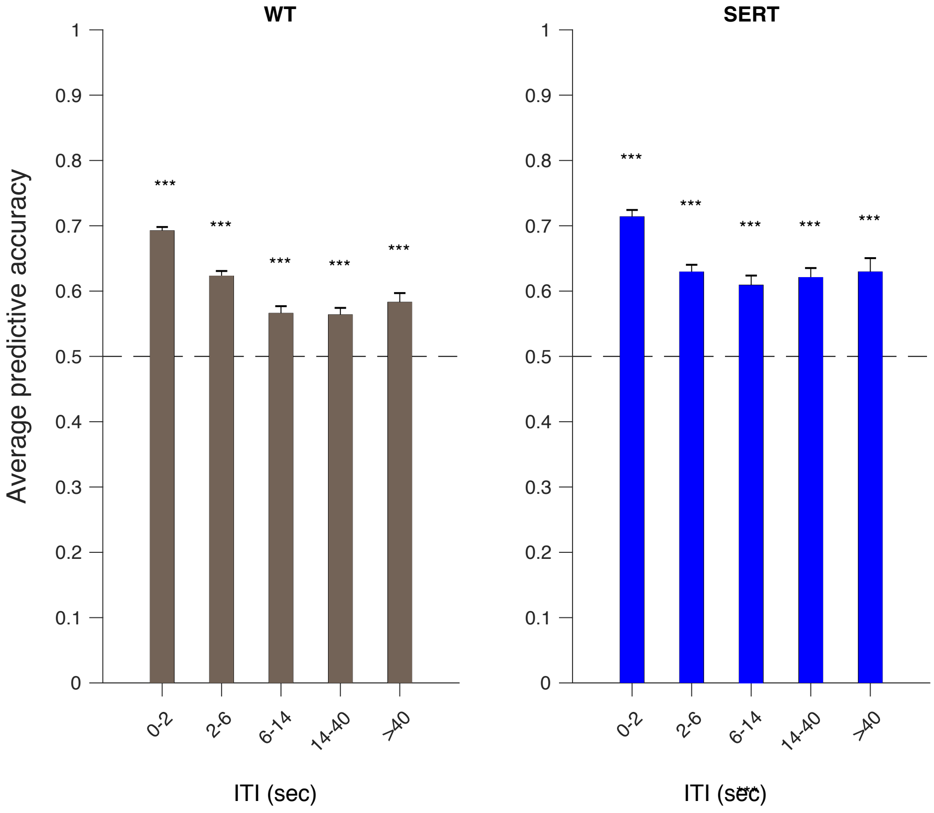

Supplementary Figure 13: Average predictive accuracies of the full model for choices following different durations of ITIs. The stars indicates the significance of the accuracy being different from chance, tested by t-tests. The error bars indicate the mean $\pm$ SEM of sessions $(n = 32)$

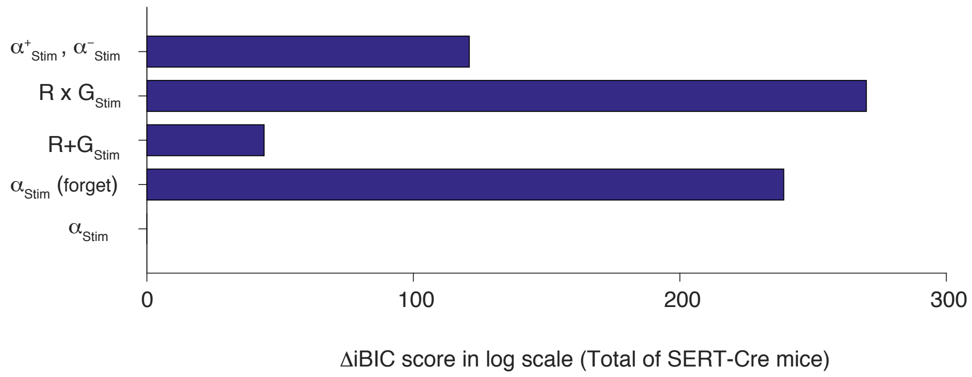

Supplementary Figure 14: Model comparison for different optogenetic stimulation effects in SERT-Cre mice, based on integrated Bayesian Information Criterion (iBIC). Our model with a modulated learning rate (bottom) outperformed models with asymmetrically modulated learning rate (top), multiplicatively modulated reward value (2nd row), and additively modulated reward value (3rd row).

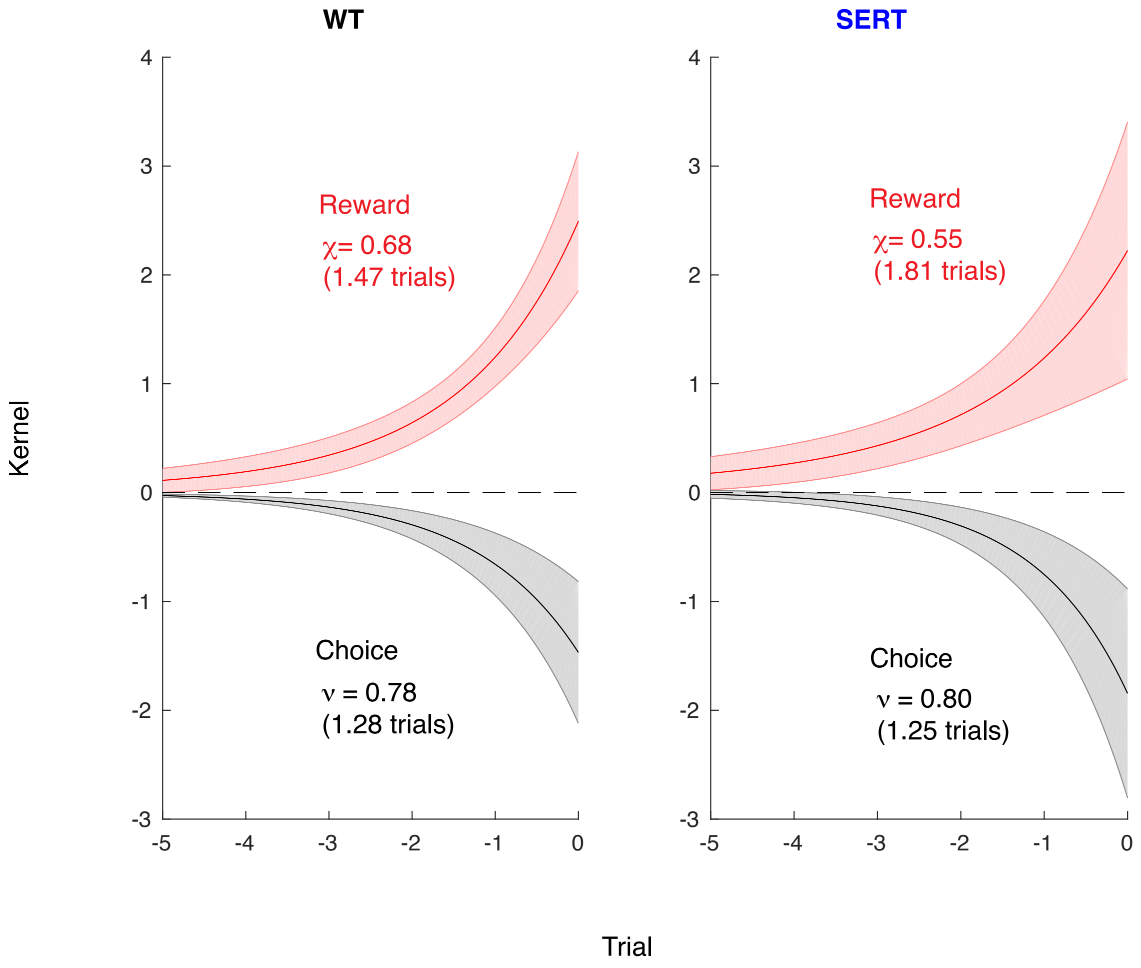

Supplementary Figure 15: Estimated choice kernel and reward kernel for the fast system in the full model. The mean $\pm$ standard deviation of estimated kernels of all traces are shown for WT (left) and SERT-Cre (right) mice. The time constants are the mean of the estimates, not the re-fit of the mean trace.

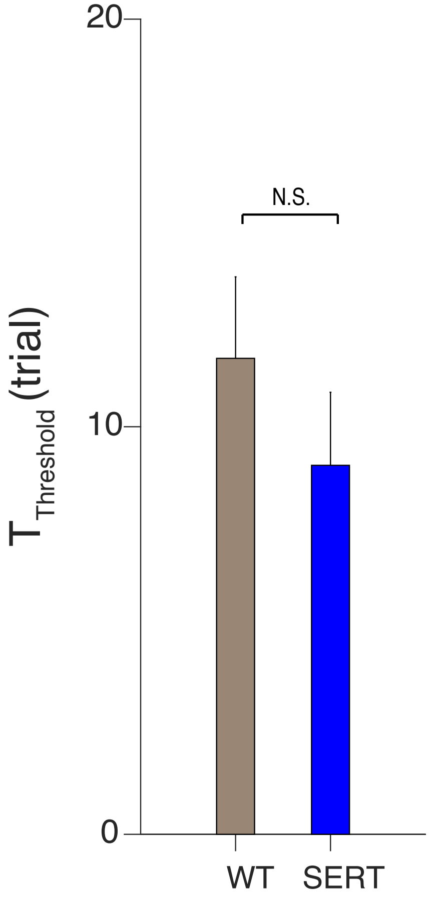

Supplementary Figure 16: Estimated threshold for the full model. The mean ± SEM of estimated kernels are shown for WT (left) and SERT-Cre (right) mice.

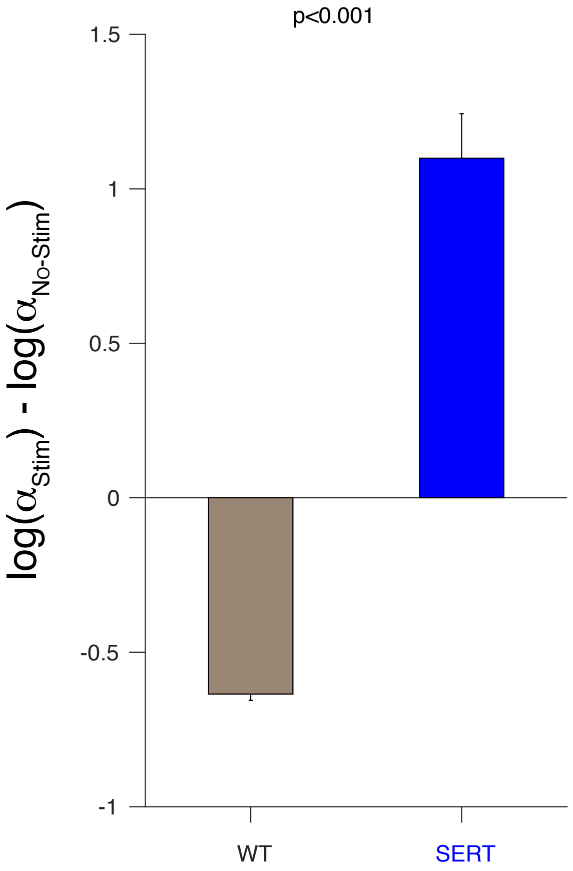

Supplementary Figure 17: [Fixed-threshold model]. Photo stimulation increased the learning rate in SERT-Cre mice. The difference between the WT mice and SERT-Cre mice was significant (permutation test, $p < 0.001$). The simple Q-learning model was assumed to learn values on all trials but was responsible for decisions on trials following long ITIs (> 7 sec). The error bars indicate the mean $\pm$ SEM of sessions ($n = 32$)

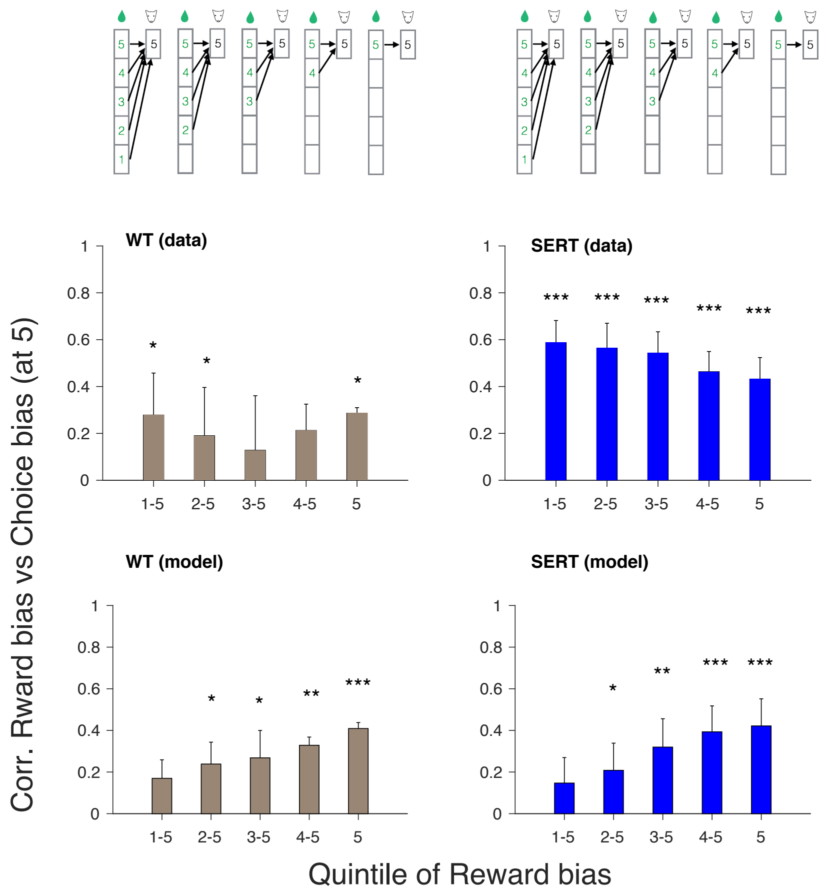

Supplementary Figure 18: Choices following long ITIs were predicted by reward history over many trials. The correlation between the choices following long ITIs in the last part of each session (5th quintile) and the reward bias estimated in different quintiles. On the x-axis, '1-5' indicates the overall reward bias computed in the total of the 1st, 2nd, 3rd, 4th and 5th quintiles, while '5' means the bias from the 5-th quintile only. The top left (top right) shows the results of WT (SERT-Cre) mice, while the bottom left (bottom right) shows the results of model's generated data for WT (SERT-Cre) mice. The stars indicate how significantly the correlation is different from zero, tested by a permutation test. The test statistic was constructed by the mean of the correlation coefficients of four animals at each quintile condition, where the correlation coefficient was computed by randomly permuted data in each condition in each animal. One star indicates $p < 0.05$; two stars indicates $p < 0.01$, while three stars indicates $p < 0.001$. The error bars indicate the mean $\pm$ SEM of sessions ($n = 32$)

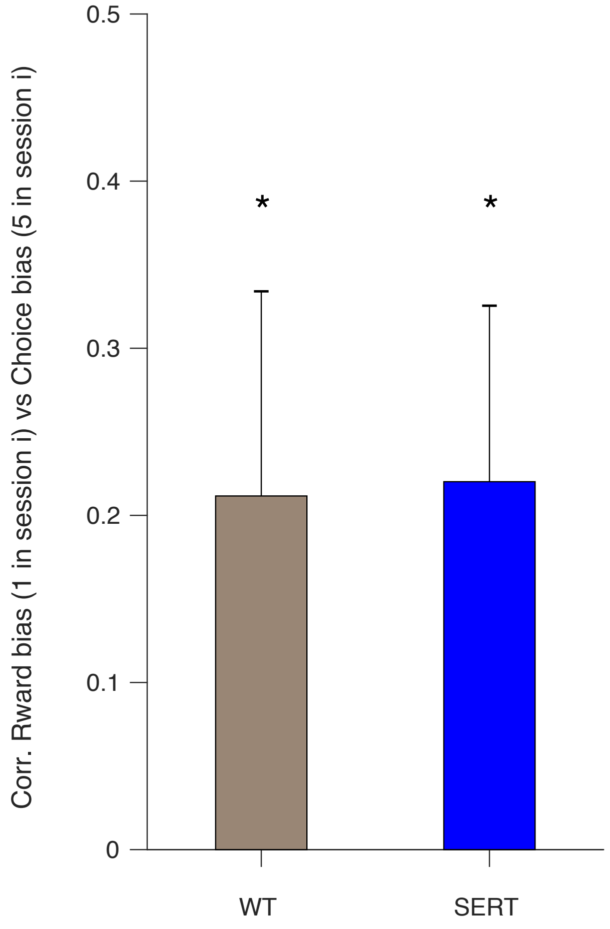

Supplementary Figure 19: Choices following long ITIs in the fifth quintile were correlated with reward bias in the first quintile in the same session. The star indicate how significantly the correlation is different from zero, tested by a permutation test. The test statistic was constructed by the mean of the correlation coefficients of four animals, where the correlation coefficient was computed by randomly permuted data in each animal. One star indicates $p < 0.05$. The error bars indicate the mean $\pm$ SEM of sessions ($n = 32$)

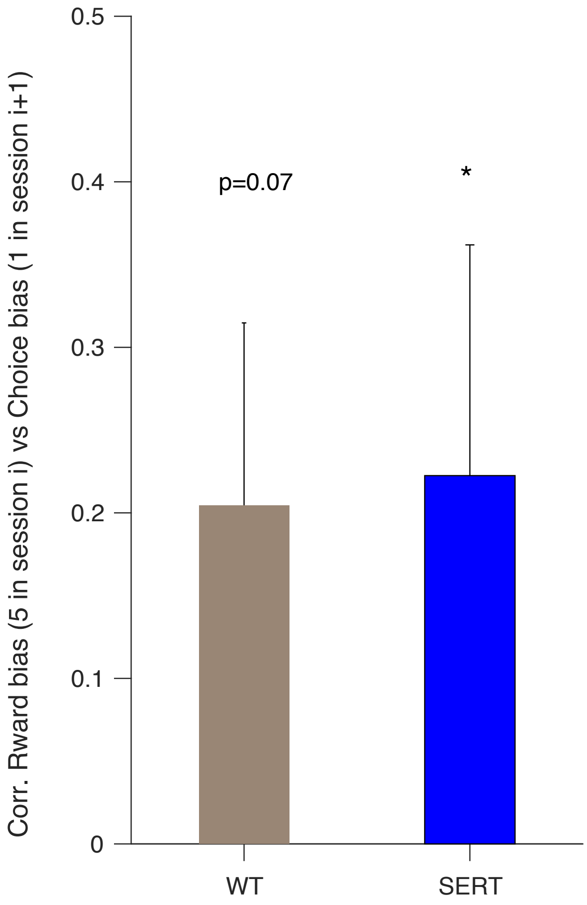

Supplementary Figure 20: Choices following long ITIs in the first quintile were correlated with reward bias in the fifth quintile in the previous session. The star indicate how significantly the correlation is different from zero, tested by a permutation test. The test statistic was constructed by the mean of the correlation coefficients of four animals, where the correlation coefficient was computed by randomly permuted data in each animal. One star indicates $p < 0.05$. The error bars indicate the mean $\pm$ SEM of sessions ($n = 28$)

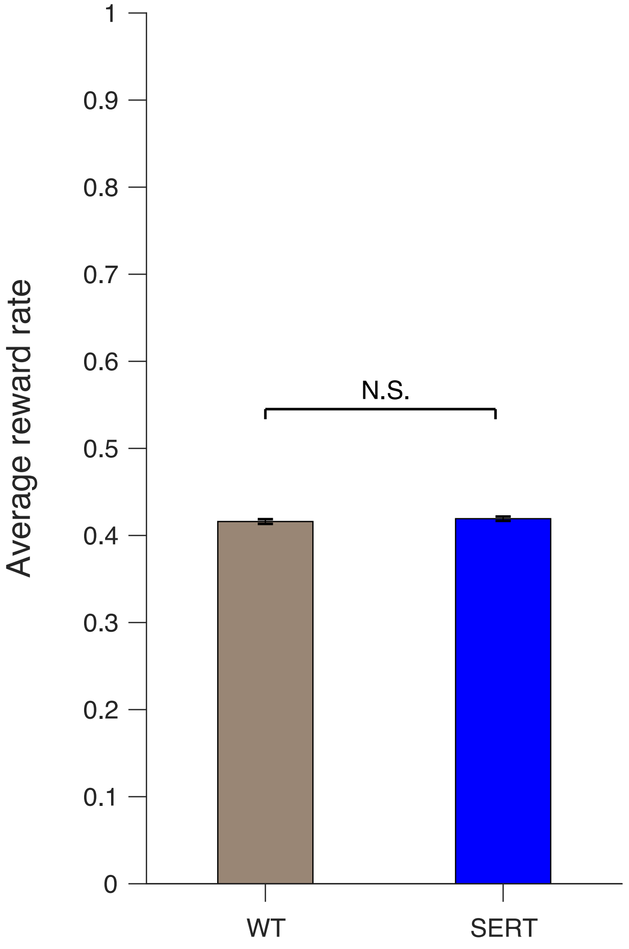

Supplementary Figure 21: Average reward rate in the task, defined by the number of rewards collected by a mouse divided by the number of trials per session. The difference between WT and SERT-Cre mice was not significant (permutation test, $p > 0.1$). The error bars indicate the mean $\pm$ SEM of sessions ($n = 32$)
